# A Sphincter-like Function of Pulmonary Vein Ostia in Normal and Atrial Fibrillation Subjects

**DOI:** 10.3390/jcdd12060203

**Published:** 2025-05-28

**Authors:** Stefano Bonapace, Matteo Falanga, Carmelo Cicciò, Cristiana Corsi, Giulio Molon

**Affiliations:** 1 Division of Cardiology, IRCCS Ospedale Sacro Cuore Don Calabria, Via Don Sempreboni 5, 37024 Negrar di Valpolicella, Italy; stefano.bonapace@sacrocuore.it; 2 Department of Electrical, Electronic and Information Engineering “Guglielmo Marconi”, University of Bologna, Campus of Cesena, 47522 Cesena, Italy; matteo.falanga2@unibo.it (M.F.); cristiana.corsi3@unibo.it (C.C.); 3 Department of Diagnostic Imaging and Interventional Radiology, IRCCS Ospedale Sacro Cuore Don Calabria, 37024 Negrar di Valpolicella, Italy; carmelo.ciccio@sacrocuore.it

**Keywords:** pulmonary veins ostia, pulmonary veins muscular sleeve, pulmonary vein sphyncterial activity, pulmonary vein atrial junction, atrial fibrillation, cardiac tomography

## Abstract

Background: Early anatomical study suggested that the pulmonary vein (PVs) junction may have putative sphincter-like activity. The aim of this study was to evaluate this activity in normal and paroxysmal/persistent atrial fibrillation subjects. Methods: The Cardiac Computed Tomography (CCT) scans of 45 subjects [15 normal controls, 15 patients with paroxysmal atrial fibrillation (PAR-AF), and 15 with persistent AF (PER-AF)] were retrospectively analyzed. All subjects were in sinus rhythm during the CCT scan. A 3D anatomical model was developed, enabling us to identify the PV ostia and to measure their area dynamic changes during the cardiac cycle. Results: The area changes in the superior PVs within the three groups were significantly higher compared to the inferior (control *p* = 0.007, PAR-AF *p* = 0.0003, PER-AF *p* = 0.04). Moreover, these variations were significantly reduced in PAR-AF and PER-AF compared to the control in all PVs (LSPV (*p* < 0.001), RSPV (*p* < 0.001), RIPV (*p* = 0.037), and LIPV (*p* < 0.001)). Conclusions: This sphincter-like activity, most prominent in superior PVs, is progressively impaired in patients with paroxysmal and persistent AF.

## 1. Introduction

Atrial fibrillation (AF) is the most common sustained cardiac arrhythmia, affecting millions of people worldwide [1,2]. AF is associated with increased risks of stroke, heart failure, overall cardiovascular morbidity, and impaired physical function [3]. Among the various triggers and mechanisms underlying AF, the pulmonary veins (PVs) play a crucial role [4]. The pulmonary veins have been identified as a major source of ectopic electrical activity that can initiate and sustain AF [4,5]. Therefore, PV ostia have received great interest due to the electrical activity of the myocardial sleeves arising from the atrial–venous junction because of atrial fibrillation (AF) ablation [4,5], while their mechanical properties have received little attention. In fact, pulmonary veins (PVs) and their atrial junctions are considered thin-walled, highly compliant, and mainly passive conduits [6]. In normal conditions, pulmonary venous blood flow is determined by the pressure gradient between the PVs and the left atrium, with a large forward flow during ventricular systole and early diastole and a small reverse flow during atrial systole [6]. Early anatomic studies in humans postulated that the myocardial fibers extending around the terminal segment of the PVs may have a putative sphincter-like function, preventing the backward flow during atrial contraction [7,8,9], but functional data confirming this mechanical activity are scarce. Interestingly, in a patient undergoing CCT scan imaging for suspected myocarditis, we observed sphincter-like contractile activity of the pulmonary veins (Video S1). We therefore decided to analyze the functional behavior of the PV ostia on a cardiac tomography scan (CT scan) in normal subjects and in patients with a history of paroxysmal and persistent atrial fibrillation to evaluate this putative sphincter-like function.

## 2. Methods

**Figure 1:**
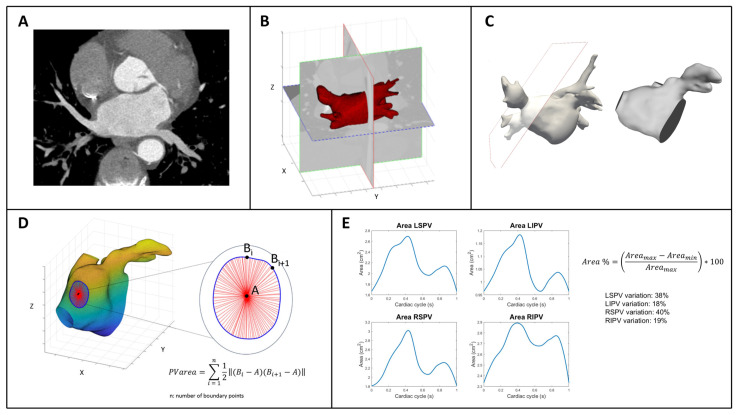
Schematic description of the workflow to analyze CT scan-derived images of PV ostia. (**A**): example of a CT plane. (**B**,**C**): examples of cut planes applied to delineate the ostia of the PVs, positioned in accordance with the anatomical ostia of the veins. (**D**): graphical explanation of the area of the PVs atrial junctions. (**E**): pulmonary veins ostia area curves throughout the entire cardiac cycle and their percentage in area variations in a normal subject. On the right, the formula to calculate the percentage area variation for each PVs ostia (see text for details). Abbreviations: PV, pulmonary vein: LSPV, left superior pulmonary vein; RSPV, right superior pulmonary vein; RIPV, right inferior pulmonary vein; LIPV, left inferior pulmonary vein.

We developed an anatomical dynamic model to segment the PV ostia derived from a contrast-enhanced CCT scan to evaluate this putative sphincter-like function in 45 subjects, 15 healthy control subjects without AF (CTRL), 15 patients with paroxysmal (PAR), and 15 patients with persistent (PER) AF referred for PV AF cryo-balloon ablation. They were included in this study if they had the most common four-PV anatomy, left (LSPV) and right (RSPV) superior PVs and left (LIPV) and right (RIPV) inferior PVs, trivial mitral regurgitation on transthoracic echocardiography, and normal ejection fraction (EF > 55%). Each patient included in this retrospective analysis provided informed consent for the data collection and analysis. All research activities adhered to the ethical guidelines outlined in the Declaration of Helsinki and received approval from the local institutional review board. Approval by the local institutional review board for the One Hospital Clinical Service was obtained in 30 June 2021. All retrospectively ECG-gated CCT scans were performed in sinus rhythm, after the intravenous administration of iodinated contrast medium, using a third-generation dual-source CT scanner (SOMATOM Force, Siemens, Erlangen, Germany). From ventricular end diastole, volumetric CCT images were reconstructed for a total of 10 phases. Each CT volume was 512 × 512 × 200 voxels. The voxel resolution was not isotropic: 0.39 mm on the x-y plane and 1 mm of slice thickness, yielding a voxel size of 0.39 × 0.39 × 1 mm^3^. Figure 1 shows the process of analyzing CT data and includes several steps that are described in this section. As shown in panel A, the first volume acquired for each patient during the cardiac cycle was segmented using an active contour algorithm featured on ITK-SNAP (version 3.8.0, Kitware Inc., Clifton Park, NY, USA). This algorithm initially focused on segmenting the LA, including the left atrial appendage (LAA), by defining a region of interest. The operator manually selected a seed, which guided the algorithm to grow towards the LA wall and enclose the entire LA anatomy, including the LAA. In order to improve the initial segmentation, its contour was regularized and smoothed using morphological operators and a Laplacian filter, respectively. Finally, the result was exported as a stereolithography file. This entire procedure was executed solely on the first obtained volume of each dynamic dataset. Through the use of Paraview (version 5.10.1, Kitware Inc.), cut planes were applied to delineate the ostia of the PVs. These planes were positioned in accordance with the anatomical ostia of the veins (Panels B and C). Because the cut alters the structure of the surface faces by removing portions of them and attached nodes, the entire surface was remeshed so that the newly established boundaries had new nodes. This was necessary to improve the performance and, consequently, the result of the next step. First, a rigid transformation was applied for initial alignment, followed by a transition to an affine registration. The result was further refined by applying a 3D non-rigid registration based on a B-spline transformation model with the mean square difference as the cost function [10,11]. The dynamic surfaces were now accessible for additional investigation for each dataset. MatlabR2023a was used to calculate the area of the PVs by loading the whole dynamic dataset of surfaces for each patient. To do so, the barycenter of each boundary was employed as a reference, as shown in Panel D. The applied formula is simply the area of the triangle as the sum of all triangles inside the boundary: *PV area* = ∑ 12 ‖(*Bi* – *A*) × (*Bi* +1 – *A*)‖ *ni* = 1, where A is the barycenter of the corresponding PV and B is the points on the boundary. This formula is applied iteratively for each PV. The final result is a PV area curve that represents the entire cardiac cycle for each patient. An example is shown in Panel E. In addition, the percentage area variation of each PV is calculated as follows: *Area*% = (*Area max* − *Area min*/*Area max*) ∗ 100.

### Statistical Analysis

Continuous variables are expressed as mean value ± standard deviation and categorical variables as percentages. For continuous variables, ANOVA was used to test differences among groups. We also conducted bivariate linear regression analyses to assess whether age or LA volume were associated with PV area variations in each vein for each study group.

## 3. Results

The mean age of the population was 60 ± 10 years, males 76%. PER were significantly older (normal 57 ± 9 years, PAR 58 ± 10 years, PER 65 ± 8 years, *p* = 0.03). No significant difference was observed in left ventricular ejection fraction among groups (normal 60.9 ± 4.3%, PAR 61.5 ± 5.4%, PER 59.7 ± 3.6%, *p* = 0.63). The echocardiographic left atrium ejection fraction (LA-EF), calculated from LA maximum and minimum volumes, was significantly lower in PER compared to normal and PAR (normal: 56.9 ± 3.5%, PAR 55.2 ± 4.2%, 51.8 ± 3.4%, *p* = 0.01). AF patients had significantly larger CT scan-measured left atrial volumes (normal 52.1 ± 16.4 mL/m^2^, PAR 62.8 ± 11.7 mL/m^2^, PER 73.5 ± 15.2 mL/m^2^, *p* = 0.002). Table 1 shows the percentage area changes in the PV ostia within the single groups; the superior PVs had significantly higher percentage area changes compared to the inferior (normal *p* = 0.007, PAR-AF *p* = 0.0003 and PER-AF *p* = 0.04) in all groups. As shown in Table 2, PV ostia areas did not differ among the three groups for LSPV (*p* = 0.75), RSPV (*p* = 0.6), and RIPV (*p* = 0.71); only LIPVs were significantly smaller in normal subjects compered to PAR-AF and PER-AF (*p* = 0.044). Notably, as shown in Table 2, percentage changes in ostia area variations were significantly reduced in PAR-AF and PER-AF compared to normal subjects in all PVs (LSPV *p* < 0.001, RSPV *p* < 0.001, RIPV *p* = 0.037 and LIPV *p* < 0.001). In the control group, LA volume was significantly associated with PV area changes in LSPV, RSPV, and RIPV (all *p* < 0.01; R^2^ ≈ 0.42–0.45), suggesting a physiological link between atrial size and PV function. In contrast, in PAR-AF and PER-AF patients, no significant associations were observed either with age or LA volume, although weak trends were noted between age and some veins (e.g., LIPV in PAR-AF, R^2^ = 0.17; *p* = 0.10).

## 4. Discussion

Our findings non-invasively confirm the presence of an active, sphincter-like contractile behavior at the junctions between the left atrium and the pulmonary veins (PVs), particularly in the superior PVs. This contractile function appears to be progressively impaired in patients with atrial fibrillation (AF).

### 4.1. Pathophysiological Considerations

Echocardiographic studies have traditionally described the PVs and their atrial junctions as thin-walled, highly compliant, and primarily passive conduits [6]. In contrast, histopathological investigations have revealed the presence of myocardial sleeves within the pulmonary veins, with more extensive extension into the superior veins compared to the inferior ones [6,8]. However, data on the mechanical activity of these myocardial sleeves remain scarce.

Nathan and Eliakim observed that myocardial fibers are often arranged in a circular or ring-like configuration around the pulmonary vein ostia [7]. These sleeves extend variably within the pulmonary veins, with greater extension in the superior veins (right superior PV: 5–25 mm; left superior PV: 8–24 mm; right inferior PV: 1–17 mm; left inferior PV: 1–19 mm) [7]. Ho and colleagues confirmed the maximum extension of the myocardium into the superior PVs to be approximately 25 mm [9]. Furthermore, Tan et al. described the muscular architecture at the veno-atrial junction as complex and less developed in the inferior PVs. They identified three distinct anatomical patterns: in pattern 1, the PV sleeves and the left atrial (LA) myocardium are disconnected; in patterns 2 and 3, there is a continuous muscular connection, albeit with differing orientations of the myocytes [12].

Gurfinkel and collaborators recorded pressure curves in both the PVs and the left atrium of patients with mitral stenosis, before and after mitral valvulotomy [8]. Their observations suggested sphincter-like activity at the PVs that failed to function when the mean LA pressure exceeded 20 mmHg. This contractile activity serves to protect the pulmonary circulation from backward pressure by impeding reverse flow. Under normal conditions, pulmonary venous blood flow is governed by the pressure gradient between the PVs and the LA, characterized by predominant forward flow during ventricular systole and early diastole, and minor reverse flow during atrial systole [6]. When LA pressure increases, compromised contractile function at the PVs contributes to increased resistance in the distal pulmonary vasculature [8]. Notably, during AF, this sphincter-like function becomes ineffective even at moderately elevated LA pressures, promoting early rises in pulmonary vascular resistance [8].

Our data support previous observations by Thiagalingam et al. [13] and Cronin et al. [14] that the behavior of the PV ostia cannot be attributed solely to passive changes in blood flow. Rather, an active contraction of the myocardial sleeves in response to passive distension—likely influenced by a Frank–Starling-like mechanism—must be considered. Importantly, while the absolute ostial area did not differ significantly between groups, we found marked differences in area variation between superior and inferior PVs. This variation aligns with the greater muscular content in the superior veins.

Moreover, we observed a progressive decline in PV ostial area variation in patients with more advanced forms of AF. This supports the notion that contractile function at the PV-LA junction is an active process that deteriorates with advancing atrial myopathy [15]. Therefore, the loss of this function may reflect the extent of atrial structural, architectural, and electrophysiological remodeling, related to fibrosis, inflammation, myocyte hypertrophy, and altered ion channel expression, which characterize atrial myopathy in AF [15], which likely extends into the PV junctions. Moreover, the fact that LA volume significantly affects PV function only in healthy subjects and not in AF patients further supports the hypothesis that the atrial myopathy related to AF is not present in normal subjects and disrupts the atrial–PV dynamics only in AF subjects.

### 4.2. Clinical Implications

Clinically, our findings suggest a shift in perspective: from viewing the PV-LA junction as a static anatomical structure to recognizing it as a dynamic, functional component. Acknowledging the progressive loss of this sphincter-like function in AF underscores the role of atrial myopathy and could refine how we stage the disease. Incorporating functional PV-LA junction assessment into pre-ablation evaluations could identify patients with early versus advanced mechanical remodeling.

For example, patients with preserved ostial contractility may respond favorably to limited PV isolation, while those with impaired function may require more extensive substrate modification. Additionally, ablation strategies could be tailored to preserve or modulate this contractile function—particularly in the superior veins, where it is most prominent—thus moving beyond purely electrical criteria for treatment.

The relevance of this mechanism may extend beyond AF. Preserving sphincter-like function could protect the pulmonary vasculature from elevated left atrial pressures, particularly in patients with heart failure. Impairment of this sphincter-like function may contribute to pulmonary hypertension, dyspnea, and right ventricular strain. Future therapies could potentially target the modulation of this function by influencing the muscular tone at these junctions.

### 4.3. Limitations

Several limitations of this study must be acknowledged: First, we have no direct anatomical characterization of the PV junction. Therefore, we were unable to categorize the anatomical patterns of the PV-LA junction in our population. This limits our ability to differentiate the relative mechanical contributions of the atrial versus PV muscular components. Second, we have no data on flow dynamics. This retrospective study did not include direct CCT measurement of pulmonary venous flow across the cardiac cycle and transthoracic echocardiography did not provide comprehensive Doppler flow data for all PVs. As a result, we could not quantify backward volume flow or precisely assess how contractile dysfunction impacts venous hemodynamics. Third, we did not consider anatomical variants. We only included patients with a normal four-vein PV anatomy. Therefore, our findings may not be generalizable to patients with anatomical variants (e.g., common trunks and additional veins), which could display different mechanical behavior. Fourth, the sample size is small. This was a pilot study with a small number of subjects. While our results are promising, they require confirmation in larger studies. Fifth, we lack longitudinal data. We did not assess patients before and after ablation or track changes in sphincter-like function over time. Longitudinal studies are needed to evaluate the impact of interventions on this contractile behavior. Seventh, although we propose a potential clinical role for PV contractility assessment, we did not correlate our imaging findings with clinical endpoints such as symptom burden, response to ablation, or long-term rhythm outcomes. Lastly, although 3D CCT modeling enabled detailed anatomical analysis, certain technical limitations may have affected the precision of ostial area measurements and functional interpretation. The workflow for the extraction of the anatomical model and its displacement field is completely automatic; therefore, it is not affected by any variability, and it was performed once per patient. Once we obtained the dynamic anatomical model, the selection of the cut planes to identify PV ostia was performed manually. This step could introduce some variability in the results, but information that could support an objective selection of the ostia is the intracardiac impedance, which is an invasive measurement not available in our population.

## 5. Conclusions

In conclusion, this pilot study introduces a novel, non-invasive approach to assess dynamic pulmonary vein ostial function using 3D CCT scan modeling. We demonstrated a sphincter-like contractile mechanism at the PV-LA junction, particularly prominent in the superior veins, that may play a protective role against backward flow during atrial contraction and is progressively impaired in AF.

## Figures and Tables

**Table 1:** PV ostia area changes within the single groups.

	LSPVs	RSPVs	RIPVs	LIPVs	* p * -Value
*PV Area changes* (%)					
Normal	33.1 ± 12.3	38.7 ± 14.6	20.3 ± 10.8	24.5 ± 10.3	0.007
Paroxysmal AF	19.4 ± 8.3	26.1 ± 8.4	13.6 ± 8.2	12.5 ± 5.3	0.0003
Persistent AF	16.0 ± 5.4	20.8 ± 10.3	12.3 ± 5.8	12.6 ± 7.0	0.04

*Abbreviations*: PVs, pulmonary veins: LSPVs, left superior pulmonary veins; RSPVs, right superior pulmonary veins; RIPVs, right inferior pulmonary veins; LIPVs, left inferior pulmonary veins.

**Table 2:** PV ostia area and PV area changes in normal, paroxysmal AF, and persistent AF.

	**Normal Subjects**	**Paroxysmal AF**	**Persistent AF**	***p*** **-Value**
*PV ostia area* (cm^2^)				
LSPVs	2.20 ± 0.59	2.19 ± 0.51	2.38 ± 1.02	0.75
RSPVs	3.20 ± 1.04	3.27 ± 1.06	3.58 ± 1.03	0.61
RIPVs	2.25 ± 0.60	2.42 ± 0.85	2.22 ± 0.63	0.71
LIPVs	1.50 ± 0.4	1.75 ± 0.06	2.06 ± 0.68	0.044
*PV area changes* (%)				
LSPVs	33.1 ± 12.3	19.4 ± 8.3	16.0 ± 5.4	<0.001
RSPVs	38.7 ± 14.6	26.1 ± 8.4	20.8 ± 10.3	<0.001
RIPVs	20.3 ± 10.8	13.6 ± 8.2	12.3 ± 5.8	0.037
LIPVs	24.5 ± 10.3	12.5 ± 5.3	12.6 ± 7.0	<0.001

*Abbreviations*: PVs, pulmonary veins: LSPVs, left superior pulmonary veins; RSPVs, right superior pulmonary veins; RIPVs, right inferior pulmonary veins; LIPVs, left inferior pulmonary veins.

## Data Availability

Data are contained within the article and Supplementary Materials.

## Supplementary Materials

The following supporting information can be downloaded at https://www.mdpi.com/article/10.3390/jcdd12060203/s1, Video S1: CT scan image of the sphincter-like movement of the 4 pulmonary veins ostia.

## Abbreviations

PV, pulmonary vein; LSPV, left superior pulmonary vein; RSPV, right superior pulmonary vein; RIPV, right inferior pulmonary vein; LIPV, left inferior pulmonary vein.
